# Evidências de Validade da Versão Brasileira da *Florida Shock Anxiety Scale* para Portadores de Cardioversor-Desfibrilador Implantável

**DOI:** 10.36660/abc.20190255

**Published:** 2020-05-22

**Authors:** Katia Regina Silva, Roberto Costa, Giovanna Regina Garcia de Oliveira Melo, Flávio Rebustini, Marcos Sidney Benedetto, Marcia Mitie Nagumo, Samuel F. Sears

**Affiliations:** 1 Instituto do Coração Faculdade de Medicina Universidade de São Paulo São PauloSP Brasil Instituto do Coração (InCor) - Faculdade de Medicina da Universidade de São Paulo,São Paulo, SP – Brasil; 2 Universidade de São Paulo Escola de Artes, Ciências e Humanidades São PauloSP Brasil Universidade de São Paulo - Escola de Artes, Ciências e Humanidades, São Paulo, SP - Brasil; 3 East Carolina University Department of Psychology and Cardiovascular Sciences Greenville North Carolina USA East Carolina University - Department of Psychology and Cardiovascular Sciences Greenville, North Carolina – USA

**Keywords:** Desfibrilador implantável, Terapias de choque, Arritmias, Ansiedade, Qualidade de vida, Validade, Confiabilidade, Psicometria

## Abstract

**Fundamento:**

A despeito da comprovada efetividade do cardioversor-desfibrilador implantável (CDI), as terapias de choque deflagradas pelo dispositivo podem causar níveis elevados de ansiedade e depressão, provocando efeitos deletérios na qualidade de vida.

**Objetivo:**

Realizar a tradução, adaptação transcultural e validação do instrumento *Florida Shock Anxiety Scale* (FSAS) para a língua portuguesa falada no Brasil.

**Métodos:**

Nesse estudo psicométrico, a validade de construto foi realizada pela análise fatorial exploratória (AFE) e confirmatória (AFC) e pela Teoria de Resposta ao Item. Os índices de ajustamento da AFC foram: Robust Mean-Scaled Chi Square/df NNFI, CFI (Comparative Fit Index), GFI (Goodness Fit Index), AGFI (Adjusted Goodness Fit Index), RMSEA (Root Mean Square Error of Approximation) e RMSR (Root Mean Square of Residuals). A confiabilidade foi verificada pelo Alfa de Cronbach, Ômega de McDonald e Greatest Lower Bound. As análises foram realizadas no SPSS 23.0 e Factor 10.8.01, com nível de significância de 5%.

**Resultados:**

A versão final em português do FSAS foi administrada em 151 portadores de CDI, com idade média de 55,7 ± 14,1 anos e predomínio do sexo masculino. A análise paralela indicou que o FSAS é unidimensional, com variância explicada de 64,4%. As correlações variaram de 0,31 a 0,77; as cargas fatoriais de 0,67 a 0,86 e as comunalidades de 0,46 a 0,74. Os índices de ajustamento da AFC estabeleceram-se acima dos limites de qualidade. Encontramos evidências satisfatórias de confiabilidade da escala FSAS.

**Conclusão:**

O instrumento FSAS-Br apresentou evidências consistentes de validade e confiabilidade, podendo, portanto, ser utilizado em portadores de CDI do Brasil. (Arq Bras Cardiol. 2020; 114(5):764-772)

## Introdução

Atualmente, não existem dúvidas quanto ao papel do cardioversor-desfibrilador implantável (CDI) na prevenção da morte súbita cardíaca, especialmente em pacientes com disfunção ventricular e doenças genéticas arritmogênicas.^1-3^Por sua comprovada eficácia na identificação e reversão de taquiarritmias ventriculares potencialmente letais, o número de implantes de CDI tem crescido exponencialmente no mundo inteiro, sendo realizados mais de 250.000 procedimentos por ano.^4^

A função primordial do CDI é reverter arritmias ventriculares potencialmente fatais pela aplicação de terapias de baixa ou de alta energia. Terapias de baixa energia, conhecidas como estimulação antitaquicardia ou sobre-estimulação, não provocam dor. Terapias de alta energia são choques de até 40 joules que, apesar de causar grande desconforto, usualmente, são aplicados após o paciente ter perdido a consciência, uma vez que são disparados cerca de 15 segundos após o início da fibrilação ventricular ou de uma taquiarritmia ventricular rápida. Em situações pouco desejáveis, como é o caso das arritmias resistentes à sobre-estimulação ou nas tempestades elétricas, choques de alta energia podem ocorrer em pacientes acordados.^3,5,6^

Estima-se que a chance de portadores de CDI necessitar de um choque apropriado para prevenção primária da morte súbita cardíaca varia de 2 a 15% ao ano.^5-8^ Por outro lado, quando o CDI é utilizado para prevenção secundária, a incidência de terapias de choque pode variar de 35 a 53%, no primeiro ano após o implante.^5-8^ A despeito do elevado nível de sofisticação tecnológica dos CDIs, lamentavelmente, existe o risco de o paciente receber um choque inapropriado em decorrência da discriminação errônea entre taquiarritmias supraventriculares e ventriculares. Nessas ocasiões, a sensação relatada é de uma experiência dolorosa e angustiante.^9-14^

Portadores de CDI convivem com a expectativa de que, a qualquer momento, o dispositivo irá aplicar terapias de choque para interromper arritmias ventriculares decorrentes de sua doença cardíaca. Desse modo, apesar de reconhecerem os benefícios do tratamento, alguns pacientes podem apresentar quadros de ansiedade, depressão, distúrbios do humor, transtorno de estresse pós-traumático, bem como medo do não funcionamento do dispositivo em momentos cruciais.^9-14^Por outro lado, tem sido reportado que o implante do CDI pode conferir aos pacientes uma grande sensação de segurança, considerando-se a capacidade do dispositivo para interromper episódios inesperados de taquiarritmias ventriculares potencialmente letais.^10-14^

Diante da preocupação com os efeitos deletérios do CDI na adaptação psicossocial, foi desenvolvido um instrumento especificamente destinado à avaliação do nível de ansiedade relacionado à presença do CDI e aos choques aplicados pelo dispositivo, para uso na prática clínica e no cenário da pesquisa científica.^15,16^ Em pouco tempo, o instrumento *Florida Shock Anxiety Scale* (FSAS) alcançou grande aceitação internacional, tendo sido traduzido e validado em diferentes culturas (Holanda,^17^ Dinamarca,^18^ Polônia,^19^ China,^20^ Noruega,^21^ Turquia^22^), com resultados consistentes.

## Objetivos

A finalidade do presente estudo foi avaliar as propriedades psicométricas da versão brasileira do instrumento FSAS para portadores de CDI.

## Métodos

### Desenho do estudo

Trata-se de um estudo transversal com duas fases: (1) Processo de tradução e adaptação transcultural do instrumento FSAS; (2) Validação de construto do instrumento FSAS.

### Local do estudo e aspectos éticos

Esse estudo foi realizado em um hospital cardiológico de alta complexidade, tendo sido aprovado pelo Comitê de Ética em Pesquisa da Instituição. Todos os participantes assinaram o termo de consentimento livre e esclarecido (TCLE).

### Instrumento Florida Shock Anxiety Scale (FSAS)

O instrumento FSAS foi desenvolvido em 2006 nos Estados Unidos para avaliar o nível de ansiedade relacionado à presença do CDI e aos choques aplicados pelo dispositivo. O instrumento consiste em 10 itens, com cinco opções de resposta (“nunca”, “quase nunca”, “de vez em quando”, “quase sempre” e “sempre”), correspondendo a uma escala do tipo Likert de 5 pontos.^15,16^

As perguntas do questionário estão relacionadas ao medo e à ansiedade dos pacientes em razão da expectativa de o dispositivo aplicar terapias de choque, bem como a mudanças comportamentais (não realizar exercícios físicos ou atividade sexual, não ficar nervoso ou com raiva, por exemplo) adotadas para evitar a ocorrência de terapias de choque, a partir da perspectiva do paciente.

O escore total do instrumento FSAS é determinado pela soma de todos os itens, podendo atingir pontuação máxima de 50 pontos. Quanto maior a pontuação, maior o nível de ansiedade, sendo que os itens que atingem três ou mais pontos devem ser considerados os aspectos mais críticos.^17,18^ O instrumento pode ser autoadministrado ou aplicado na forma de entrevista.

### Fase 1 - Tradução e adaptação transcultural do instrumento

O processo de adaptação transcultural do instrumento FSAS seguiu as recomendações internacionais e constou de cinco etapas: (1) Tradução por dois colaboradores independentes; (2) Consolidação das traduções pelo comitê de especialistas; (3) Retrotradução; (4) Harmonização das versões pelo comitê de especialistas; (5) Pré-teste com a população alvo; (6) Revisão do pré-teste e versionamento final.^23-25^ O comitê de especialistas foi constituído por profissionais da área da estimulação cardíaca artificial e enfermeiros.

**Figura 1:**
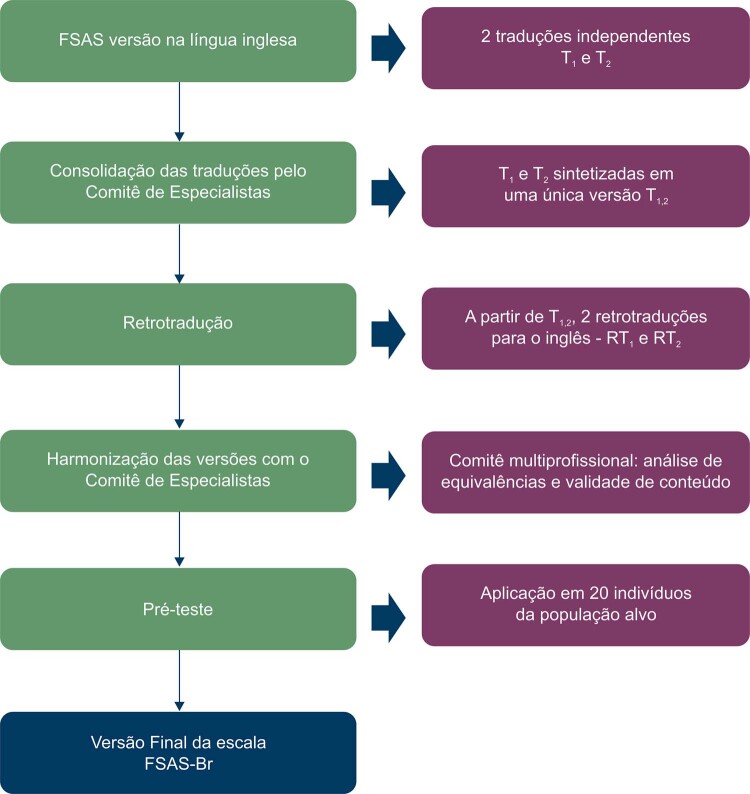
– *Processo de adaptação transcultural do instrumento FSAS.*

A tradução do instrumento original para a língua portuguesa foi realizada por dois colaboradores independentes, brasileiros com proficiência na língua portuguesa e inglesa. Nessa etapa, as versões elaboradas pelos dois tradutores independentes (T_1_ e T_2_) foram consolidadas em uma única versão (T_1-2_), após discussão com o comitê de especialistas. A partir dessa versão traduzida, foi realizada a etapa da retrotradução (RT_1_ e RT_2_) por dois professores bilíngues que não tinham tido contato prévio com o instrumento original. A finalidade dessa etapa foi aferir a consistência semântica e idiomática das traduções realizadas na primeira etapa. Finalmente, foi realizada uma nova reunião com o comitê de especialistas para revisar todo o processo de adaptação transcultural e realizar a harmonização das versões, obtendo-se, assim, a versão pré-final do instrumento. (Figura 1)

A versão pré-teste foi administrada em uma amostra de conveniência de 20 participantes, com idades entre 18 e 80 anos, portadores de CDI por pelo menos seis meses e que realizavam acompanhamento clínico em nossa instituição. O pré-teste foi realizado para identificar e corrigir possíveis problemas de tradução. Após o preenchimento do instrumento pelo próprio indivíduo, realizou-se uma entrevista de esclarecimento, para verificar se havia itens de difícil compreensão ou irrelevantes, assim como aferir a compreensão de cada item do instrumento. O critério estabelecido para revisão e modificação da tradução foi a compreensão dos itens por menos de 80% dos entrevistados.

### Fase 2 – Validação de construto do instrumento FSAS

A versão final em português do instrumento FSAS foi administrada em uma amostra de conveniência de 151 participantes com as seguintes características: (1) adultos, com idades entre 18 a 80 anos, de ambos os sexos e com qualquer nível de escolaridade; (2) portadores de CDI há mais de seis meses; (3) capacidade de compreender e responder às perguntas do questionário utilizado no estudo; (4) concordância em participar do estudo, com assinatura do TCLE. Não foram incluídos no estudo os indivíduos que apresentaram ao menos uma das seguintes situações: (1) indicação de transplante cardíaco; (2) gestação em curso; (3) neoplasias malignas.

Os pacientes foram selecionados, de forma consecutiva, durante o atendimento ambulatorial ou mediante visitas realizadas à Unidade de Internação da nossa instituição. Os indivíduos que preencheram os critérios de elegibilidade foram convidados a responder o questionário FSAS. Nesse mesmo momento, era realizado o levantamento do histórico clínico e obtidas informações sobre o implante do CDI, terapias de choque prévias e uso de medicamentos.

Todos os dados foram coletados diretamente em *tablets*, utilizando-se formulários eletrônicos desenvolvidos no sistema *Research Electronic Data Capture* (REDCap)^26^ hospedado no servidor da nossa instituição.

#### Número amostral

O dimensionamento do número de participantes nos estudos psicométricos usualmente é feito sobre o número de itens dos instrumentos avaliados. Alguns estudos demonstraram que uma relação de 20:1 ou superior, ou seja, 20 participantes para cada item do instrumento, seria o ideal. Contudo, relações de 10:1 já permitem análises adequadas. Desse modo, foi estabelecido o mínimo amostral de 150 pacientes.^25^

## Análise Estatística

### Análise descritiva

Foi realizada uma análise descritiva minuciosa utilizando medidas de tendência central (média, desvio padrão, mediana, média aparada, intervalos de confiança e intervalo interquartil). O teste de Kolmogorov-Smirnov (KS) foi utilizado para a testagem isolada da normalidade de cada item do questionário, enquanto que o Teste de Mardia foi empregado para verificar a normalidade multivariada.

Todas as análises foram realizadas nos softwares SPSS 23.0 e Factor 10.8.01, adotando-se o nível de significância de 5%.

### Validade de construto e dimensionalidade

Neste estudo, foram realizadas as análises fatoriais exploratória (AFEs) e confirmatória (AFC) para verificar a dimensionalidade do instrumento FSAS em sua versão na língua portuguesa.

A testagem da dimensionalidade foi realizada pela Análise Paralela Robusta (APR), por meio da *Optimal Implementation of Parallel Analysis* (PA), com minimum rank factor analysis (MRFA), que minimiza a variância comum dos resíduos.^27,28^A robustez do teste foi determinada a partir da associação de um *bootstrap* com uma extrapolação amostral para 5000. A extração dos fatores foi feita inicialmente com RULS (*Robust Unweighted Least Squares*), que reduz os resíduos das matrizes.^29^

### Teoria de Resposta ao Item

Foi utilizado o índice de discriminação do item (a), que mede a força de associação entre o item e a variável latente e tem interpretação similar às cargas fatoriais da AFE.

### Parâmetros de qualidade da versão traduzida e adaptada do instrumento FSAS

Para a adequação dos itens e do modelo, foram considerados os seguintes critérios: variância explicada do modelo (60 a 70%), valores de cargas fatoriais (> 0,50), comunalidades (> 0,40) e discriminação do item, problemas de colinearidade e multicolinearidade (cargas fatoriais acima de 0,80 e 0,85, respectivamente).

### Índices de ajustamento na Análise Fatorial Confirmatória

Os índices de ajustamento do modelo e os respectivos valores esperados foram: *Robust Mean-Scaled Chi Square/df NNFI* (*Non-Normed Fit Index* > 0,93), CFI (*Comparative Fit Index* > 0,94), GFI (*Goodness Fit Index* > 0,95), AGFI (*Adjusted Goodness Fit Index* > 0,93), RMSEA (*Root Mean Square Error of Approximation* < 0,07) e RMSR (*Root Mean Square of Residuals* < 0,08).29-31

### Confiabilidade

Foram adotados três indicadores para a avaliação da confiabilidade da versão brasileira do questionário FSAS: Alfa de Cronbach, Ômega de McDonald e GLB (*Greatest Lower Bound*).

## Resultados

### Versão final do instrumento FSAS

De um modo geral, as etapas de tradução e adaptação transcultural produziram versões semelhantes do instrumento FSAS. A síntese das traduções foi bem concisa e uniu o mais coerente de cada tradução realizada. As versões produzidas na retrotradução evidenciaram uma boa qualidade das traduções e do processo de síntese realizado nas etapas iniciais.

Participaram do pré-teste 20 portadores de CDI, com idade média de 55,6 ± 6,8 anos, dos quais 50% eram do sexo feminino, 50% eram brancos e 30% tinham cursado até o Ensino Médio. Todos os participantes consideraram os itens relevantes, de fácil entendimento e compreenderam as opções de respostas. Nenhum participante sugeriu modificações no instrumento. Na Tabela 1 estão apresentados os itens do instrumento em suas versões na língua inglesa e portuguesa.

**Tabela 1:** – Instrumento original e versão brasileira da Florida Shock Anxiety Scale (FSAS-Br)

Item	Instrumento Original	Versão Brasileira: FSAS-Br
1	I am scared to exercise because it may increase my heart rate and cause my device to fire.	Eu tenho medo de fazer exercícios físicos porque isso pode aumentar meus batimentos cardíacos e fazer o meu CDI me aplicar um choque.
2	I am afraid of being alone when the ICD fires and I need help.	Eu tenho medo de estar sozinho e precisar de ajuda quando o CDI me aplicar um choque.
3	I do not get angry or upset because it may cause my ICD to fire.	Eu não posso ficar nervoso ou chateado porque isso pode fazer o CDI me aplicar um choque.
4	It bothers me that I do not know when the ICD will fire.	Me sinto preocupado por não saber quando o CDI vai me aplicar um choque.
5	I worry about the ICD not firing sometime when it should.	Eu me preocupo com a possibilidade do CDI não funcionar quando eu precisar.
6	I am afraid to touch others for fear I’ll shock them if the ICD fires.	Eu tenho medo de tocar nas pessoas e dar um choque nelas caso o CDI dispare.
7	I worry about the ICD firing and creating a scene.	Eu me preocupo sobre a possibilidade de assustar as pessoas quando o CDI me aplicar um choque.
8	When I notice my heart beating rapidly, I worry that the ICD will fire.	Quando eu percebo que meu coração bate mais rápido, eu fico preocupado que o CDI vai me aplicar um choque.
9	I have unwanted thoughts of my ICD firing.	Eu penso o tempo todo que a qualquer momento o CDI pode me aplicar um choque.
10	I do not engage in sexual activities because it may cause my ICD to fire.	Eu não tenho relações sexuais porque isso pode fazer o CDI me aplicar um choque.
	Response options Not at all Rarely Some of the time Most of the time All the time	Opções de resposta Nunca Quase nunca Algumas vezes Na maioria das vezes Sempre

### Propriedades psicométricas do instrumento FSAS

#### Composição da População

Nesta fase do estudo, foram incluídos 151 portadores de CDI, com idade média de 55,7±14,1 anos (variação de 19 a 80 anos). Houve predomínio do sexo masculino que correspondeu a 64% dos casos. A maioria dos pacientes era da raça branca (85,4%) e 49% haviam cursado o ensino fundamental (Tabela 2).

**Tabela 2:** – Perfil demográfico e clínico dos participantes do estudo

Características	
Sexo masculino	64,0%
Idade (anos)	55,7 ± 14,1
Raça branca	85,4%
**Escolaridade**	
Ensino Superior	14,8%
Ensino Médio	34,9%
Ensino Fundamental	49,0%
Analfabeto	1,3%
**Estado civil**	
Casado	64,9%
Solteiro	14,6%
Divorciado	7,9%
Viúvo	6,6%
União estável	6,0%
**Doença cardíaca estrutural**	
Doença de Chagas	30,5%
Cardiopatia isquêmica	25,2%
Cardiopatia hipertrófica	14,6%
Cardiopatia dilatada	13,2%
Síndrome de Brugada	4,6%
Síndrome do QT longo congênita	3,3%
Displasia arritmogênica do ventrículo direito	2,6%
2Outras	5,9%
**Classe Funcional da New York Heart Association**	
I	37,1%
II	47,7%
III	11,3%
IV	4,0%
Fração de ejeção do ventrículo esquerdo (ecocardiograma)	41,2 ± 15,6
**Comorbidades**	
Nenhuma	29,1%
Hipertensão arterial	49,5%
Doença arterial coronariana	15,9%
Diabetes	20,6%
Fibrilação atrial	27,1%
Insuficiência renal crônica	6,5%
Dislipidemia	51,4%
Índice de comorbidades de Charlson	1,3 ± 1,0
**Uso de medicamentos**	
IECA/ BRA	72,7%
Betabloqueadores	85,4%
Diuréticos	50,7%
Antiarrítmicos	58,9%
Antiagregantes plaquetários	31,8%
Anticoagulantes orais	27,8%
**Indicação do CDI**	
Prevenção primária da morte súbita	19,9%
Prevenção secundária da morte súbita	80,1%
**Tipo de CDI**	
CDI ventricular	41,1%
CDI atrioventricular	46,4%
CDI com ressincronizador cardíaco	12,6%
Tempo de implante do CDI (anos)	6,7 ± 4,4
**Terapias do CDI**	
Recebeu terapias de choque	60,3%
Nunca recebeu terapias de choque	39,7%

*BRA: bloqueador do receptor de angiotensina; IECA: Inibidor da enzima de conversão da angiotensina*

Dentre as doenças cardíacas, chama a atenção a Doença de Chagas, que esteve presente em 30,5% dos casos, seguida pela cardiopatia isquêmica em 25,2%. A Síndrome de Brugada e a Síndrome do QT longo congênita foram identificadas em 4,6 e 3,3% dos pacientes, respectivamente.

A avaliação basal mostrou que a maioria dos pacientes estava em classe funcional I (37,1%) e II (47,7%), segundo os critérios da *New York Heart Association*. A função ventricular esquerda determinada pelo ecocardiograma transtorácico bidimensional variou de 18 a 77%, com mediana de 35%.

Apenas 29,1% dos pacientes não apresentavam comorbidades associadas. Dislipidemia e hipertensão arterial foram as comorbidades mais frequentes, estando presentes em 51,4% e 49,5% dos pacientes, respectivamente. Fibrilação atrial estava presente em 27,1% dos indivíduos estudados (Tabela 2).

Como já era esperado, 80,1% das indicações do CDI corresponderam à profilaxia secundária da morte súbita arrítmica, visto que, na realidade brasileira, devido à escassez de recursos, o implante de CDI ainda é pouco utilizado para profilaxia primária da morte súbita.

Quanto ao tipo de CDI, sistemas unicamerais estavam presentes em 41,1% dos pacientes, seguidos pelos sistemas dupla-câmara (46,4%). Apenas 12,6% dos pacientes apresentavam CDI com ressincronizador cardíaco, por terem doença cardíaca mais avançada. O tempo de uso do CDI foi de 6,7 ± 4,4 anos, variando de 6 meses a 16,9 anos. O número expressivo de pacientes (60,3%) que haviam recebido terapias de choque do CDI foi condizente com a indicação clínica do dispositivo, visto que terapias de choque são muito mais frequentes em indivíduos submetidos à profilaxia secundária da morte súbita.

## Análise descritiva dos itens do instrumento FSAS

Através da análise descritiva dos itens do instrumento, foi possível identificar que houve violação da normalidade da distribuição, indicando, portanto, a necessidade da utilização de correlações policóricas, ao invés da correlação de Pearson.

As médias dos itens do instrumento variaram de 1,5 a 2,9. O escore médio do FSAS foi de 22,8 ± 11,1, com mediana de 20 pontos e variação de 10 a 50 pontos. Não houve efeito de valores extremos na média (Tabela 3).

**Tabela 3:** – Análise descritiva dos itens da FSAS-Br

Item	Média	DP	Limite inferior	Limite superior	5% da média aparada	Mediana	Amplitude	IQ	Assimetria	Curtose	K-S	Sig.
1	2,95	1,86	2,66	3,25	2,95	3,00	4,00	4,00	0,12	-4,80	0,29	0,01
2	2,46	1,72	2,19	2,74	2,40	1,00	4,00	4,00	2,84	-3,67	0,33	0,01
3	2,26	1,69	1,99	2,53	2,18	1,00	4,00	3,00	4,06	-2,90	0,37	0,01
4	2,47	1,69	2,20	2,74	2,41	1,00	4,00	3,00	2,63	-3,69	0,33	0,01
5	2,43	1,62	2,17	2,69	2,37	2,00	4,00	3,00	2,97	-3,21	0,30	0,01
6	1,54	1,25	1,34	1,74	1,38	1,00	4,00	0,00	10,81	7,67	0,48	0,01
7	2,36	1,68	2,09	2,63	2,29	1,00	4,00	3,00	3,34	-3,30	0,34	0,01
8	2,74	1,72	2,47	3,02	2,71	3,00	4,00	4,00	1,30	-4,14	0,28	0,01
9	2,07	1,59	1,81	2,32	1,96	1,00	4,00	2,00	5,37	-1,56	0,39	0,01
10	1,54	1,24	1,34	1,74	1,37	1,00	4,00	0,00	10,87	7,84	0,49	0,01

## Validade de construto e dimensionalidade do instrumento FSAS

Os valores obtidos do índice de Kaiser-Meyer-Olkin (KMO= 0,88), do teste de esfericidade de Bartlett (X^2^= 565,5, df= 45; p< 0,001) e o determinante da matriz (0,0206 (p<0,0001)) revelaram uma correlação significativa entre os itens, confirmando a adequação da aplicação da AFE.

A análise paralela indicou a existência de apenas uma dimensão para o instrumento. Ademais, esse conjunto de itens consegue explicar 64,4% da variável latente (acima dos valores recomendados na literatura). ^29-31^ O conceito dos autovalores (eigenvalue > 1) também apontou apenas uma dimensão com um autovalor de 6,08. O fato de o instrumento ser unidimensional dispensou a necessidade de técnicas rotacionais da matriz fatorial. A unidimensionalidade indicou o uso da técnica *Normal-ogive graded response model* para a TRI, mais adequada para modelo unidimensional politômico.^31^

A tabela 4 apresenta as cargas fatoriais, que variaram de 0,67 a 0,86, representando níveis excelentes de aderência dos itens à variável latente, acima do critério mínimo de 0,50, sem indícios de multicolinearidade. A unidimensionalidade eliminou a possibilidade de dupla saturação (*cross-loading*). As comunalidades variaram de 0,46 a 0,74, com todos os itens acima do limite de 0,40. Para a discriminação do item (a), os valores variaram de 0,91 a 1,71, também demonstrando boa aderência à variável latente e confirmando os dados obtidos através das cargas fatoriais.

**Tabela 4:** – Validade de construto da FSAS-Br: cargas fatoriais, comunalidades, discriminação do item

Item	Cargas fatoriais	Comunalidades (h^2^)	Discriminação do item (a)
1	0,76	0,58	1,17
2	0,77	0,60	1,22
3	0,76	0,59	1,19
4	0,81	0,65	1,37
5	0,68	0,46	0,93
6	0,71	0,50	1,00
7	0,67	0,46	0,91
8	0,73	0,53	1,05
9	0,86	0,74	1,71
10	0,74	0,55	1,11

A AFC revelou um bom ajuste ao modelo unidimensional, com valores semelhantes aos recomendados pela literatura: *Robust Mean and Variance-Adjusted Chi Square* X^2^/ df (35) = 40,40; p < 0,243; NNFI= 0,997; CFI= 0,997; GFI= 0,986; AGFI= 0,982. Os indicadores de resíduos ficaram em níveis bons (RMSEA= 0,032; RMSR= 0,077), indicando pouca diferença entre a matriz original e a matriz gerada pela carga dos fatores.^31^

## Confiabilidade do instrumento FSAS-Br

Encontramos evidências satisfatórias de confiabilidade da escala FSAS-Br, com coeficiente alfa de Cronbach de 0,92, Ômega de McDonald de 0,92 e GLB de 0,98.

## Discussão

No presente estudo, foi descrito o processo de tradução e adaptação transcultural de uma escala de avaliação do nível de ansiedade relacionado às terapias de choque do desfibrilador implantável, atendendo ao rigor metodológico preconizado na literatura internacional.^23-25^ A versão final da escala FSAS para a língua portuguesa (FSAS-Br) falada no Brasil, apresentou equivalência conceitual, semântica, cultural e de mensuração em relação aos itens originais no inglês.^15,16^

Esforços foram empreendidos para incluir pacientes com diferentes perfis sociodemográficos e diferentes tipos de doença cardíaca para garantir uma representação heterogênea, visando assegurar a melhor calibração dos itens do instrumento. Desse modo, pacientes com diferentes tipos de CDI (ventricular, atrioventricular ou associado à terapia de ressincronização cardíaca) foram incluídos, assim como pacientes com indicação de profilaxia primária ou secundária da morte súbita cardíaca. Não obstante, os principais grupos de cardiopatias comuns a esse perfil de pacientes também foram contemplados, com expressiva prevalência da Doença de Chagas, cardiopatia isquêmica e hipertrófica.

No cenário internacional, a escala FSAS tem sido amplamente utilizada em diferentes contextos, já que apresenta boa sensibilidade para identificar o nível de ansiedade relacionado às terapias de choque do CDI e tempo reduzido para preenchimento.^15-22^ Desse modo, é importante ressaltar que a escala FSAS não foi projetada para avaliar aspectos relevantes da adaptação ao dispositivo e o seu real impacto na qualidade de vida, fazendo-se necessária a utilização de outros instrumentos para complementar a avaliação desses pacientes. Nesse sentido, os autores da escala FSAS desenvolveram outro instrumento, o *Florida Patient Acceptance Survey* (FPAS),^32^ que visa avaliar a adaptação psicossocial dos portadores de dispositivos cardíacos implantáveis. Os resultados do processo de adaptação transcultural e validação para a língua portuguesa da escala FPAS serão publicados oportunamente.

A comprovação das evidências de validade de um instrumento tem sido recomendada pela comunidade científica como forma de verificar se o instrumento realmente mensura a variável latente de interesse com precisão. Além disso, é importante analisar se a estrutura fatorial do instrumento está adequadamente representada pela sua dimensionalidade, ou seja, o número de dimensões que compõem o instrumento de avaliação.^27-31^ Na publicação original do FSAS, os autores postulam que o instrumento é bidimensional, apresentando uma dimensão composta por 7 itens (“*Consequence*”) e outra com 3 itens (“*Trigger*”).^15^ Esse modelo não foi reprodutível para a versão brasileira, visto que todas as análises realizadas no presente estudo sustentaram a unidimensionalidade da escala FSAS-Br. Revisitando o estudo de Kuhl et al.,15 é importante ressaltar que a amostra foi constituída por apenas 72 participantes, o que pode ter causado impacto nos resultados das análises psicométricas.

Posteriormente, a escala FSAS foi submetida a um processo de avaliação de suas propriedades psicométricas, dessa vez, com uma casuística de 443 participantes.16 A AFC mostrou que as duas dimensões anteriormente identificadas eram altamente correlacionadas a um fator de segunda ordem (“*Shock anxiety*”), ou seja, as duas dimensões identificadas previamente poderiam ser melhor explicadas pela sua associação a um fator comum, que seria a dimensão “Ansiedade relacionada ao choque”. Em virtude desses resultados, os autores passaram a recomendar a utilização da pontuação total da escala FSAS, ao invés de subdividi-la nas duas dimensões anteriormente descritas. Esses resultados corroboraram a estrutura fatorial identificada em nosso estudo.

As análises de confiabilidade da escala FSAS-Br revelaram a precisão da versão brasileira, que foi confirmada pelos valores adequados do coeficiente alfa de Cronbach, Ômega de McDonald e GLB. A adoção desses três indicadores buscou aumentar a precisão da interpretação, visto que o coeficiente alfa de Cronbach sofre efeitos da natureza da distribuição dos dados e do tamanho da amostra. Além disso, seus valores podem estar elevados em decorrência de escalas extensas, de itens paralelos e/ou redundantes ou da cobertura restrita do construto em análise, tornando a medida de confiabilidade frágil.^33^

De um modo geral, os resultados observados no presente estudo demonstraram que o instrumento é confiável e válido para aplicação no Brasil, atendendo adequadamente aos critérios de qualidade propostos para medidas de desfechos reportados por pacientes.

### Limitações do estudo

Embora a população estudada seja maior do que as amostras de várias publicações anteriores que utilizaram a escala FSAS, a condução de novos estudos com amostras mais robustas é fundamental para a consolidação de sua validade e para atestar sua estabilidade nos mais diversos cenários e perfis de pacientes com CDI.

Estudos futuros, analisando a associação dos escores da FSAS-Br com a ocorrência de terapias de choque do CDI e outros parâmetros clínicos, serão úteis para identificar fatores que podem estar associados aos níveis elevados de ansiedade e, assim, permitir o estabelecimento de intervenções específicas e personalizadas para esses pacientes.

## Conclusões

O instrumento FSAS-Br apresentou evidências consistentes de validade e confiabilidade, podendo-se, portanto, recomendar sua utilização na população de portadores de CDI do Brasil, na prática clínica e em pesquisas científicas.
