# Supplementary material for: Evidências de Validade da Versão Brasileira da Florida Shock Anxiety Scale para Portadores de Cardioversor-Desfibrilador Implantável
Source: Arq Bras Cardiol. 2020 May 22;114(5):764–72. [Article in Portuguese] doi: 10.36660/abc.20190255 (PMC8387007; doi:10.36660/abc.20190255)
Supplement: Translation

# Translation: Validity Evidence of the Brazilian Version of the Florida Shock Anxiety Scale for Patients with Implantable Cardioverter Defibrillators

**Authors:** Katia Regina Silva, Roberto Costa, Giovanna Regina Garcia de Oliveira Melo, Flávio Rebustini, Marcos Sidney Benedetto, Marcia Mitie Nagumo, Samuel F. Sears

**Affiliations:** 1 Brazil Instituto do Coração (InCor) - Faculdade de Medicina da Universidade de São Paulo, São Paulo, SP – Brazil; 2 Brazil Universidade de São Paulo - Escola de Artes, Ciências e Humanidades, São Paulo, SP - Brazil; 3 USA East Carolina University - Department of Psychology and Cardiovascular Sciences Greenville, North Carolina – USA

**Keywords:** Implantable defibrillator, Shock therapies, Arrhythmias, Anxiety, Psychometric

## Abstract

**Background:**

In spite of proven effectiveness of implantable cardioverter defibrillators (ICDs), shock therapy delivered by the device may result in increased levels of anxiety and depression, leading to deleterious effects on quality of life.

**Objective:**

To carry out the translation, cross-cultural adaptation and validation of the *Florida Shock Anxiety Scale* (FSAS) scale into Brazilian Portuguese.

**Methods:**

In this psychometric study, construct validity was performed by exploratory (EFA) and confirmatory (CFA) factor analyses, and by item response theory (IRT). The adjustment indexes of the CFA were: Robust Mean-Scaled Chi Square/df NNFI, CFI (Comparative Fit Index), GFI (Goodness Fit Index), AGFI (Adjusted Goodness Fit Index), RMSEA (Root Mean Square Error of Approximation) and RMSR (Root Mean Square of Residuals). Reliability was evaluated through Cronbach’s Alpha, McDonald’s Omega and Greatest Lower Bound (GLB). The analyses were carried out with the programs SPSS 23 and Factor 10.8.01. A 5 percent significance level was used.

**Results:**

The final Portuguese version of the FSAS was administered to 151 ICD patients, with a mean age of 55.7 ± 14.1 years, and predominantly male. The parallel analysis indicated that the FSAS is unidimensional, with an explained variance of 64.4%. The correlations ranged from 0.31 to 0.77, factor loadings from 0.67 to 0.86, and communalities from 0.46 to 0.74. The adjustment indexes of the CFA were above the quality threshold. Satisfactory reliability evidence was provided by the FSAS.

**Conclusions:**

The FSAS-Br showed consistent validity and reliability evidence. Therefore, it can be used in ICD patients in Brazil. (Arq Bras Cardiol. 2020; 114(5):764-772)

## Introduction

Nowadays, there are no doubts regarding the role of the implantable cardioverter defibrillator (ICD) for prevention of sudden cardiac death, especially among patients with ventricular dysfunction and arrhythmogenic genetic diseases.^1-3^ Due to its proven efficacy in identifying and correcting potentially lethal ventricular tachyarrhythmias, the number of ICD implantations has increased significantly worldwide, and more than 250,000 procedures are performed every year.^4^

The primary purpose of ICD is to correct potentially fatal ventricular arrhythmias by delivering low- or high-energy therapy. Low-energy therapy, known as antitachycardia pacing or antitachycardia pacing (ATP), is a painless method. High-energy therapy delivers shocks of up to 40 J which, in spite of causing major discomfort, usually occur after the patient has lost consciousness, since they are applied about 15 seconds after the initiation of ventricular fibrillation or fast ventricular tachyarrhythmia. In undesirable situations, such as arrhythmias resistant to overstimulation, or in electrical storm, high-energy discharges can occur in awake patients.^3,5,6^

It is estimated that the chances of ICD patients will need appropriate electric shocks for primary prevention of sudden cardiac death varies between 2 and 15% per year.^5-8^ On the other hand, when the ICD is used for secondary prevention, the incidence of shock therapies may vary between 35 and 53%, within the first year after implantation.^5-8^ Despite the high level of technological sophistication of ICDs, unfortunately, there is the risk that the patient may receive inappropriate shock deliveries as a result of erroneous discrimination between supraventricular and ventricular tachyarrhythmias. On these occasions, the sensation reported is a painful and distressing experience.^9-14^

ICD patients live with the expectation that, at any moment, the device will deliver shock therapies to interrupt ventricular arrhythmias resulting from their heart disease. Thus, although they recognize the benefits of the treatment, some patients may present with anxiety, depression, mood disorders, post-traumatic stress disorder, as well as fear that the device will not operate in crucial situations.^9-14^ On the other hand, ICD implantation has been reported to provide the patient with a great sense of safety, considering the device’s capacity to interrupt unexpected episodes of potentially fatal ventricular arrhythmias.^10-14^

In face of the concern about the deleterious effects of ICD on psychosocial adaptation, an scale was specifically developed to assess the level of anxiety related to the presence of ICD and to the shocks delivered by the device, for use both in clinical practice and in the context of scientific research.^15,16^ The Florida Shock Anxiety Scale (FSAS) quickly achieved wide international acceptance, and has been translated and validated in several countries (Netherlands,^17^ Denmark,^18^Poland,^19^ China,^20^ Norway,^21^ Turkey^22^), with consistent results.

## Objectives

The purpose of the present study was to assess the psychometric properties of the Brazilian version of the FSAS for ICD patients.

## Methods

### Study design

This study was conducted in a high-complexity cardiology hospital and it was approved by that hospital’s Committee of Ethics in Research. All subjects signed a free and informed consent form.

#### Study location and ethical aspects

This was a psychometric study of cross-cultural adaptation and validation of the FSAS.

## The Florida Shock Anxiety Scale (FSAS)

The FSAS was developed in 2006 in the United States to provide a quantitative measure of ICD shock-related anxiety. The instrument consists of 10 items, with five response options (“not at all”,”rarely”,”some of the time”,”most of the time”,”all the time”), corresponding to a 5-point Likert scale.^15,16^

Questions are related to patients’ fear or anxiety caused by the expectation that the device may deliver shock therapies and to the behavioral changes (not engaging in physical exercise or in sexual activity, or not getting angry or upset, for instance) to avoid the occurrence of ICD therapies.

The FSAS total score is determined by the sum of all items, with a maximum score of 50 points. The higher the score, the higher the anxiety level. The items receiving three points or more should be considered the most critical aspects.^17,18^The instrument can be self-administered or administered by interview.

## Stage 1 – Translation and cross-cultural adaptation of the instrument

The cross-cultural adaptation process of the FSAS followed international guidelines and included five stages: (1) Translation by two independent translators; (2) Synthesis of the translations; (3) Back-translation; (4) Harmonization of the translations by the expert committee; (5) Pretest with the target population; (6) Pretest review and final translation.^23-25^The expert committee was composed by professionals of the area of artificial cardiac stimulation and by nurses.

The translation of the original instrument into Portuguese was performed by two independent Brazilian translators, proficient both in the Portuguese and English languages. In this stage, the translations produced by the two independent translators (T_1_ and T_2_) were reconciled into one version (T_1_-_2_), after discussion with the expert committee. Working from the final version and blind to the original version, two bilingual teachers carried out the back-translations (RT_1_ and RT_2_). The aim of this stage was to measure the semantic and idiomatic consistency of the translations produced in the first stage. Finally, a new meeting was held with the expert committee to review all the cross-cultural adaptation process and undertake the harmonization across versions, thus obtaining the pre-final version of the instrument (Figure 1).

**Figure 1:** – *Cross-cultural adaptation process of the FSAS instrument*

The pretest version was administered in a convenience sample of 20 ICD patients, aged between 18 and 80 years, All patients were recruited during an outpatient cardiovascular clinic appointment, and were at least 6 months postimplant. The pretest was conducted to identify and correct possible translation problems. Following self-completion of the instrument, a clarifying interview was held to verify the existence of irrelevant or hardly understandable items, as well as to measure the understanding of each item of the instrument. It was established that the translation would be reviewed or reformulated if less than 80% of the participants were able to understand the items.

## Stage 2 – construct validation of the FSAS

The final Portuguese version of the FSAS was administered to a convenience sample of 151 participants with the following characteristics: (1) adults, aged between 18 and 80 years, of both sexes and with any education level; (2) ICD implanted for more than 6 months; (3) capable of understanding and answering the questionnaire used in the study; (4) having agreed to participate in the study by signing the informed consent form. We did not include in the study patients presenting at least one of the following situations: (1) indication for cardiac transplantation; (2) ongoing pregnancy; (3) malignant neoplasia.

Patients were selected consecutively, during outpatient care or by visits to the inpatient unit of our institution. Individuals who met the eligibility criteria were invited to answer the FSAS questionnaire. At the same time, demographic, clinical and ICD data were collected by using electronic case report forms developed in REDCap^26^ (Research Electronic Data Capture) hosted at the hospital’s server.

### Sample size

Sample size determination for psychometric studies is usually calculated based on the number of items of the instrument. Some studies have demonstrated that a ratio of 20:1 or greater, that is, 20 participants per item would be ideal. However, ratios of 10:1 are sufficient to allow for adequate analysis. Thus, a minimum sample number of 150 patients was established.^25^

## Statistical Analysis

### Descriptive analysis

Detailed descriptive analysis was performed, using measures of central tendency (mean, standard deviation, median, trimmed average, confidence intervals and interquartile interval). The Kolmogorov-Smirnov (KS) test was used to test the normality of each item in the questionnaire, whereas the Mardia test was employed to assess multivariate normality.

All analyses were performed using SPSS 23 statistical package software and Factor 10.8.01, adopting a level of significance of 5%.

### Construct validity and dimensionality

In this study, we conducted an exploratory factor analysis (EFA) and a confirmation factor analysis (CFA) to verify the dimensionality of the FSAS in its Portuguese version.

The dimensionality testing was performed using Robust Parallel Analysis (RPA) through the Optimal implementation of Parallel Analysis (PA) with minimum rank factor analysis (MRFA), which minimizes the common variance of residuals.^27,28^The robustness of the test was determined from the association of a bootstrap with sample extrapolation to 5,000. Factor extraction was done initially with Robust Unweighted Least Squares (RULS), which reduces the matrix of residuals.^29^

### Item Response Theory

Item discrimination index was used (a), which measures the association strength between the item and the latent variable, and whose interpretation is similar to factor loading in the exploratory factor analysis.

### Quality parameters of the translated and adapted versions of the FSAS

To adequate the items and the models, the following criteria were taken into account: the explained variance of the model (60 to 70%), factor loading values (> 0.50), communalities (> 0.40) and item discrimination, and collinearity and multicollinearity problems (factor loads ranging from 0.80 to 0.85).

### Indices of adjustment obtained in the Confirmatory Factor Analysis

The model adjustment indices and their respective expected values were: Robust Mean-Scaled Chi Square/df NNFI (Non-Normed Fit Index > 0.93), CFI (Comparative Fit Index > 0.94), GFI (Goodness Fit Index > 0.95), AGFI (Adjusted Goodness Fit Index > 0.93), RMSEA (Root Mean Square Error of Approximation < 0.07) and RMSR (Root Mean Square of Residuals < 0.08).^29-31^

### Reliability

Three indicators were adopted to assess the reliability of the Brazilian version of the FSAS questionnaire: Coefficient Alpha (“Cronbach’s Alpha”), Omega and the Greatest Lower Bound (GLB).

## Results

### The final version of the FSAS

The stages of the translation and cross-cultural adaptation resulted in similar versions of the FSAS instrument. The synthesis of the translations was quite concise and combined the most coherent elements of each translation. The back-translations confirmed the good quality of the translations and the synthesis process carried out in the initial stages.

A total of 20 ICD patients, with a mean of age 55.6 ± 6.8 years, took part in the pretest. Of these, 50% were female, 50% were white and 30% had studied up to High School. All participants reported that the items were relevant, easy to understand and that the response options were clear. No modifications in the instrument were required. Table 1 shows the instrument items in its English and Portuguese versions.

**Table 1:** – Original and Brazilian version of the Florida Shock Anxiety Scale (FSAS-Br) instrument

Item	Original Instrument	Brazilian version: FSAS-Br
1	I am scared to exercise because it may increase my heart rate and cause my device to fire.	Eu tenho medo de fazer exercícios físicos porque isso pode aumentar meus batimentos cardíacos e fazer o meu CDI me aplicar um choque.
2	I am afraid of being alone when the ICD fires and I need help.	Eu tenho medo de estar sozinho e precisar de ajuda quando o CDI me aplicar um choque.
3	I do not get angry or upset because it may cause my ICD to fire.	Eu não posso ficar nervoso ou chateado porque isso pode fazer o CDI me aplicar um choque.
4	It bothers me that I do not know when the ICD will fire.	Me sinto preocupado por não saber quando o CDI vai me aplicar um choque.
5	I worry about the ICD not firing sometime when it should.	Eu me preocupo com a possibilidade do CDI não funcionar quando eu precisar.
6	I am afraid to touch others for fear I’ll shock them if the ICD fires.	Eu tenho medo de tocar nas pessoas e dar um choque nelas caso o CDI dispare.
7	I worry about the ICD firing and creating a scene.	Eu me preocupo sobre a possibilidade de assustar as pessoas quando o CDI me aplicar um choque.
8	When I notice my heart beating rapidly, I worry that the ICD will fire.	Quando eu percebo que meu coração bate mais rápido, eu fico preocupado que o CDI vai me aplicar um choque.
9	I have unwanted thoughts of my ICD firing.	Eu penso o tempo todo que a qualquer momento o CDI pode me aplicar um choque.
10	I do not engage in sexual activities because it may cause my ICD to fire.	Eu não tenho relações sexuais porque isso pode fazer o CDI me aplicar um choque.
	Response options Not at all Rarely Some of the time Most of the time	Opções de resposta Nunca Quase nunca Algumas vezes Na maioria das vezes

### Psychometric properties of the FSAS

#### Population composition

In this stage of the study, 151 ICD patients, with a mean of 55.7±14.1 years (range, 19- 80 years), were included. There was a male sex predominance, which corresponded to 64% of the cases. Most patients were white (85.4%) and 49% had attended Middle School (Table 2).

**Table 2:** – Demographic and clinical profile of the study participants

Characteristics	
Male sex	64.0%
Age (years)	55.7 ± 14.1
White	85.4%
**Education**	
Higher Education	14.8%
High School	34.9%
Middle School	49.0%
Illiterate	1.3%
**Marital Status**	
Married	64.9%
Single	14.6%
Divorced	7.9%
Widow	6.6%
Stable union	6.0%
**Structural Heart Disease**	
Chagas Disease	30.5%
Ischemic Cardiomyopathy	25.2%
Hypertrophic Cardiomyopathy	14.6%
Dilated Cardiomyopathy	13.2%
Brugada syndrome	4.6%
Congenital Long QT Syndrome	3.3%
Right Ventricular Arrhythmogenic Dysplasia	2.6%
Others	5.9%
**New York Heart Association Functional Class**	
I	37.1%
II	47.7%
III	11.3%
IV	4.0%
Left Ventricular Ejection Fraction (Echocardiography)	41.2 ± 15.6
**Comorbidities**	
None	29.1%
Hypertension	49.5%
Coronary Artery Disease	15.9%
Diabetes	20.6%
Atrial Fibrillation	27.1%
Chronic Kidney Disease	6.5%
Dislipidemia	51.4%
Charlson comorbidity index	1.3 ± 1.0
**Use of medication**	
ACEI/ARB	72.7%
Beta blockers	85.4%
Diuretics	50.7%
Antiarrhythmic drugs	58.9%
Platelet antiaggregants	31.8%
Oral anticoagulants	27.8%
**ICD indication**	
Primary prevention of sudden cardiac death	19.9%
Secondary prevention of sudden cardiac death	80.1%
**ICD Type**	
Ventricular ICD	41.1%
Atrioventricular ICD	46.4%
Cardiac resynchronization ICD	12.6%
Time of ICD implantation (years)	6.7 ± 4.4
**ICD therapies**	
Received shock therapies	60.3%
Never received shock therapies	39.7%

*ARB: angiotensin receptor blocker; ACEI: angiotensin-converting enzyme inhibitor.*

Among the cardiac diseases, there was a predominance of Chagas disease, which was present in 30.5% of the cases, followed by ischemic cardiomyopathy in 25.2%. Brugada syndrome and congenital long-QT syndrome (LQTS) were identified in 4.6 and 3.3% of patients, respectively.

Baseline assessment showed that most patients were in the New York Heart Association (NYHA) functional classes I (37.1%) and II (47.7%). Left ventricular function was determined by bidimensional transthoracic echocardiography and ranged from 18 to 77%, with a median of 35%.

Only 29.1% of the patients did not present any associated comorbidities. Dyslipidemia and arterial hypertension were the most frequent comorbidities, being present in 51.4% and 49.5% of patients, respectively. Atrial fibrillation was present in 27.1% of the individuals studied (Table 2).

As expected, 80.1% of the indications for ICD were r secondary prophylaxis of sudden cardiac death. In Brazil, due to lack of resources, ICD implantation is still underused for primary prophylaxis of sudden cardiac death.

## Descriptive analysis of the FSAS items

Through descriptive analysis of the instrument items, it was possible to identify that normality of distribution was violated, indicating, therefore, the need for polychoric correlation, instead of Pearson’s correlation coefficient.

The means of the instrument items ranged from 1.5 to 2.9. The FSAS average score was 22.8 ± 11.1, with a median of 20 points and variation of 10 to 50 points. There was no impact of extreme values on the mean (Table 3).

**Table 3:** – Descriptive analysis of the FSAS-Br items

Item	Average	SD	Inferior threshold	Superior threshold	5 % trimmed average	Median	Range	IQR	Asymmetry	Kurtosis	KS	Sig.
1	2.95	1.86	2.66	3.25	2.95	3.00	4.00	4.00	0.12	-4.80	0.29	0.01
2	2.46	1.72	2.19	2.74	2.40	1.00	4.00	4.00	2.84	-3.67	0.33	0.01
3	2.26	1.69	1,99	2,53	2,18	1,00	4,00	3,00	4,06	-2,90	0,37	0,01
4	2,47	1,69	2,20	2,74	2,41	1,00	4,00	3,00	2,63	-3,69	0,33	0,01
5	2,43	1,62	2,17	2,69	2,37	2,00	4.00	3.00	2.97	-3.21	0.30	0.01
6	1.54	1.25	1.34	1.74	1.38	1.00	4.00	0.00	10.81	7.67	0.48	0.01
7	2.36	1.68	2.09	2.63	2.29	1.00	4.00	3.00	3.34	-3.30	0.34	0.01
8	2.74	1.72	2.47	3.02	2.71	3.00	4.00	4.00	1.30	-4.14	0.28	0.01
9	2.07	1.59	1.81	2.32	1.96	1.00	4.00	2.00	5.37	-1.56	0.39	0.01
10	1.54	1.24	1.34	1.74	1.37	1.00	4.00	0.00	10.87	7.84	0.49	0.01

## Construct validity and dimensionality of the FSAS

The values obtained from the Kaiser-Meyer-Olkin index (KMO= 0.88), the Bartlett’s sphericity test (X^2^= 565.5, df= 45; p<0.001) and the matrix determinant (0.0206 (p<0.0001)) revealed a significant correlation between the items, which confirmed the adequacy of the EFA.

The parallel analysis indicated the existence of only one dimension for the instrument. Moreover, this item set can explain 64.4% of latent variable (above the values recommended in the literature).^29-31^ The eigenvalue criteria also indicated only one dimension, with a an eigenvalue of of 6.08. The fact that the instrument was unidimensional waived requirements for methods of matrix factor rotation. Unidimensionality indicated the use of the normal-ogive graded response IRT model, which is more adequate for a unidimensional polytomous model.^31^

Table 4 presents the factor loads, which ranged from 0.67 to 0.86, representing excellent levels of adherence of the items to the latent variable, greater than the minimum criterion of 0.50, with no evidence of multicollinearity. The unidimensionality ruled out the possibility of cross-loading. Communalities varied between 0.46 and 0.74, with all the items above the threshold of 0.40. For item discrimination (a), the values ranged from 0.91 to 1.71, also indicating good adherence to the latent variable and corroborating the data obtained from factor loading.

**Table 4:** – Construct validity of the FSAS-Br: factor loading, communalities and item description

Item	Factor loading	Communalities (h^2^)	Item description (a)
1	0.76	0.58	1.17
2	0.77	0.60	1.22
3	0.76	0.59	1.19
4	0.81	0.65	1.37
5	0.68	0.46	0.93
6	0.71	0.50	1.00
7	0.67	0.46	0.91
8	0.73	0.53	1.05
9	0.86	0.74	1.71
10	0.74	0.55	1.11

The CFA revealed good adjustment to the unidimensional model, with values similar to those recommended by the literature: Robust Mean and Variance-Adjusted Chi Square X^2^/ df (35) = 40.40; p < 0.243; NNFI= 0.997; CFI= 0,997; GFI= 0.986; AGFI= 0.982. The residual indicators were at good levels (RMSEA= 0.032; RMSR= 0.077), showing little difference between the original matrix and the matrix generated from factor loadings.^31^

## Reliability of the FSAS-Br

Satisfactory reliability evidence was provided by the FSAS-Br scale, with a Cronbach’s alpha coefficient of 0.92, a McDonald’s Omega coefficient of 0.92 and GLB of 0.98.

## Discussion

In the present study, we described the translation and cross-cultural adaptation process of a brief scale designed to provide a quantitative measure of ICD shock-related anxiety, folllowing international methodological standards.^23-25^ The final translation of the FSAS into Brazilian Portuguese (FSAS-Br) presented conceptual, semantic, cultural and measurement equivalences compared to the original items in English.^15,16^

Efforts were made to include patients with different sociodemographic profiles and various types of underlying heart diseases to ensure heterogeneous representation, aiming at providing the best calibration of the items. Thus, patients with different ICD types (ventricular, atrioventricular or associated with cardiac resynchronization therapy) were included, as well as patients with indications for primary or secondary prophylaxis of sudden cardiac death. Notwithstanding, the most common kinds of heart disease among these patients’ profiles have also been contemplated, with expressive prevalence of Chagas Disease, ischemic and hypertrophic cardiomyopathy.

In the international scenario, the FSAS scale has been widely used in different scenarios, since it presents good sensitivity to identify the level of ICD shock-related anxiety and requires reduced time for completion.^15-22^ Thus, it is important to highlight that the FSAS was not designed to assess relevant aspects of adaptation to the device and its real impact on quality of life, which makes it necessary to use other instruments to complement the assessment of these patients. In this sense, the authors who had created the FSAS developed another instrument, the Florida Patient Acceptance Survey (FPAS),^32^ which aims at assessing the psychosocial adjustment of ICD patients. The results of the cross-cultural adaptation and validation process of the FPAS into Portuguese will be published in due course.

Evidence of validity of an instrument has been recommended by the scientific community as a way to check whether the instrument actually and accurately measures the latent variable of interest. In addition, it is important to analyze whether the instrument factor structure is adequately represented by its dimensionality, that is, the number of dimensions that make up the instrument of assessment.^27-31^ In the original publication of the FSAS, the authors claim that the instrument was bidimensional, presenting two dimensions: Consequence (composed of 7 items) and Trigger (composed of 3 items).^15^This model was not reproducible to the Brazilian version, because all analyses performed in this study supported the FSAS-Br scale unidimensionality. Revisiting the study by Kuhl et at.,^15^ it is important to highlight that the sample was constituted by only 72 participants, which may have had an impact on the results of the psychometric analyses.

Afterwards, the psychometric properties of the FSAS were evaluated, with a sample of 443 participants.^16^ The CFA showed that the two previously identified dimensions were highly related to a second-order factor (“Shock anxiety”). In other words, the two dimensions identified previously could have been better explained by their association to a common factor, namely the “shock-related anxiety” dimension. Due to these results, the authors recommended that the total scale score may be more clinically useful, instead of subdividing it into the two dimensios described before. These results corroborate the factor structure identified in our study.

Reliability assessment of the FSAS-Br scale revealed the accuracy of the Brazilian version, which was confirmed by adequate values of Cronbach’s alpha, McDonald’s Omega and GLB. The adoption of these three indications aimed to increase the accuracy of interpretation, since the Cronbach’s coefficient alpha is affected by the nature of data distribution and by sample size. Besides, its values may be increased by extensive scales, parallel and/or redundant elements or limited coverage of the construct under analysis, decreasing the reliability of the measurement.^33^

In general, the results observed in the present study showed that the instrument is reliable and valid for application in Brazil, meeting the quality requirements for patient-reported outcome measurements.

### Study limitations

Although the population studied is larger than the samples of several other studies which have used the FSAS, further studies with more robust samples are crucial for the consolidation of its validity and for attesting its stability in the various possible scenarios and profiles of ICD patients.

Further studies, evaluating the association of the FSAS-Br scores with the occurrence of ICD shock therapies and other clinic parameters will be useful to identify factors which may be associated with increased anxiety levels and, therefore, allow for the establishment of specific and personalized interventions for these patients.

## Conclusions

The FSAS-Br instrument presented consistent validity and reliability evidence and, therefore, its use can be recommended for the ICD population in Brazil, both in clinical practice and in scientific research.
